# Supplementary material for: C-terminal truncation modulates α-Synuclein’s cytotoxicity and aggregation by promoting the interactions with membrane and chaperone
Source: Commun Biol. 2022 Aug 9;5:798. doi: 10.1038/s42003-022-03768-0 (PMC9363494; doi:10.1038/s42003-022-03768-0)
Supplement: Supplementary file 2 — Supplementary Information [file 42003_2022_3768_MOESM2_ESM.pdf]

C-terminal truncation modulates α-Synuclein's cytotoxicity and aggregation by promoting the interactions with membrane and chaperone

Cai Zhang[1,2#], Yunshan Pei[1,2#], Zeting Zhang[1,3*], Lingling Xu[1], Xiaoli Liu[1], Ling Jiang[1,3], Gary J Pielak[4], Xin Zhou[1,3], Maili Liu[1,2,3], Conggang Li[1,2,3*]

[1]Key Laboratory of Magnetic Resonance in Biological Systems, State Key Laboratory of Magnetic Resonance and Atomic and Molecular Physics, National Center for Magnetic Resonance in Wuhan, Wuhan Institute of Physics and Mathematics, Innovation Academy for Precision Measurement Science and Technology, Chinese Academy of Sciences, 430071, Wuhan, China. [2]Graduate University of Chinese Academy of Science, 100049, Beijing, China. [3]Wuhan National Laboratory for Optoelectronics, Huazhong University of Science and Technology, 430071, Wuhan, China. [4]Department of Chemistry, Department of Biochemistry and Biophysics, Lineberger Comprehensive Cancer Center, Integrative Program for Biological and Genome Sciences, University of North Carolina, Chapel Hill, NC 27599, USA.

[#] These authors contributed equally to this work.

*Corresponding author: Zeting Zhang, Conggang Li

Email: conggangli@apm.ac.cn; zhangzeting@apm.ac.cn

Supplementary Figure 1

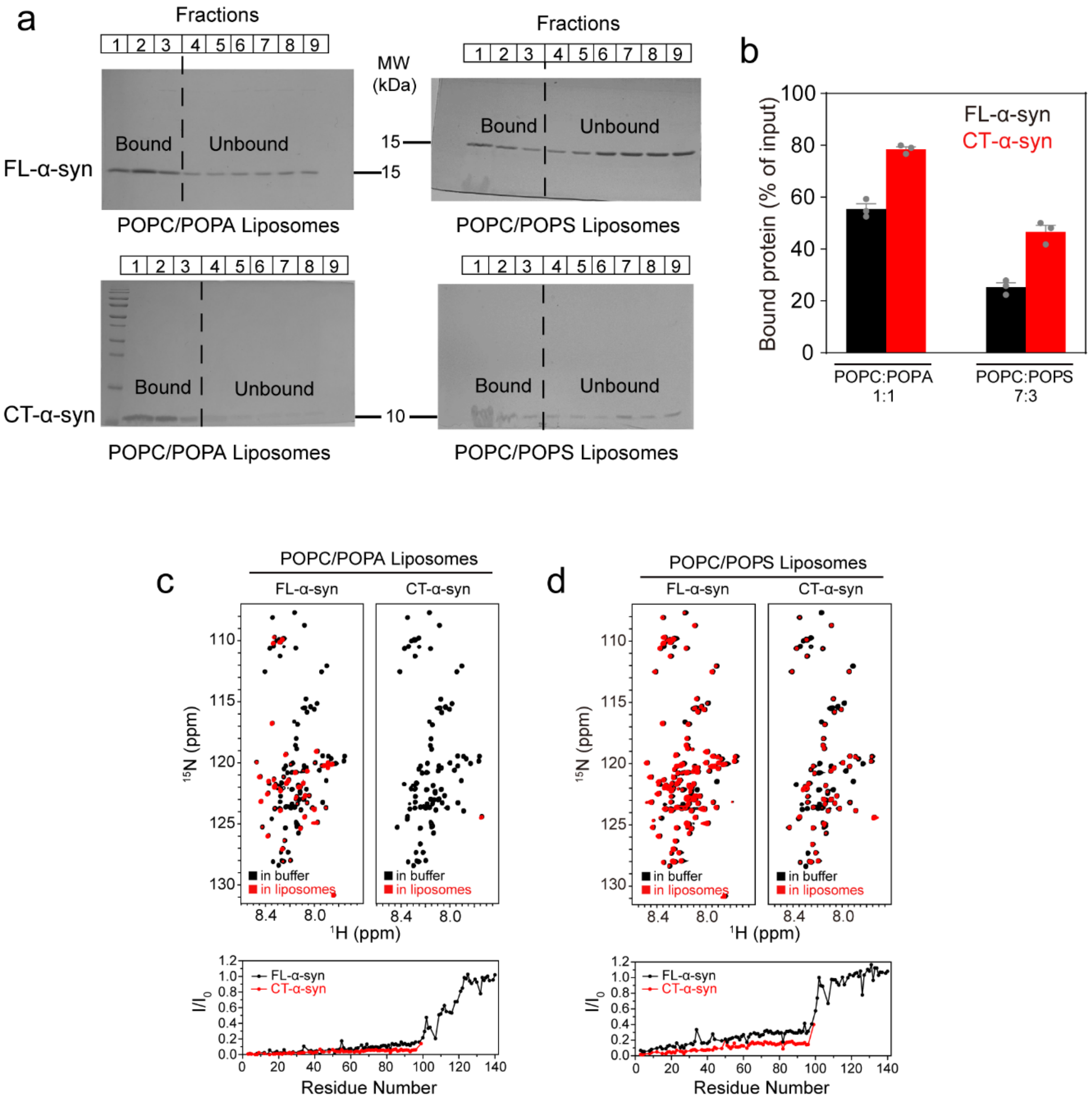

Stronger interaction of CT-α-syn with membranes. (a) SDS polyacrylamide gels of post gradient-centrifugation fractions using liposomes containing 50% POPA and 30% POPS, respectively. FL- and CT-α-syn samples were run on SDS-PAGE respectively, and lipids migrated out of the gel as much as possible. (b) Percent lipid-bound protein from SDS-PAGE. Uncertainties are the standard error of the mean from three independent trials. (c-d) Overlay of ^{1}H-^{15}N HSQC spectra of 0.125 mM ^{15}N-enriched FL- and CT-α-syn in the absence (black) and presence (red) of 12 mM POPC/POPA (1:1) liposomes (c) and POPC/POPS (7:3) liposomes (d). Residue-resolved NMR signal attenuation (I/I_0) of FL- (black circles) and CT-α-syn (red circles) upon adding liposomes below the corresponding ^{1}H-^{15}N HSQC spectra. Values<1.0 indicate interaction.

Supplementary Figure 2

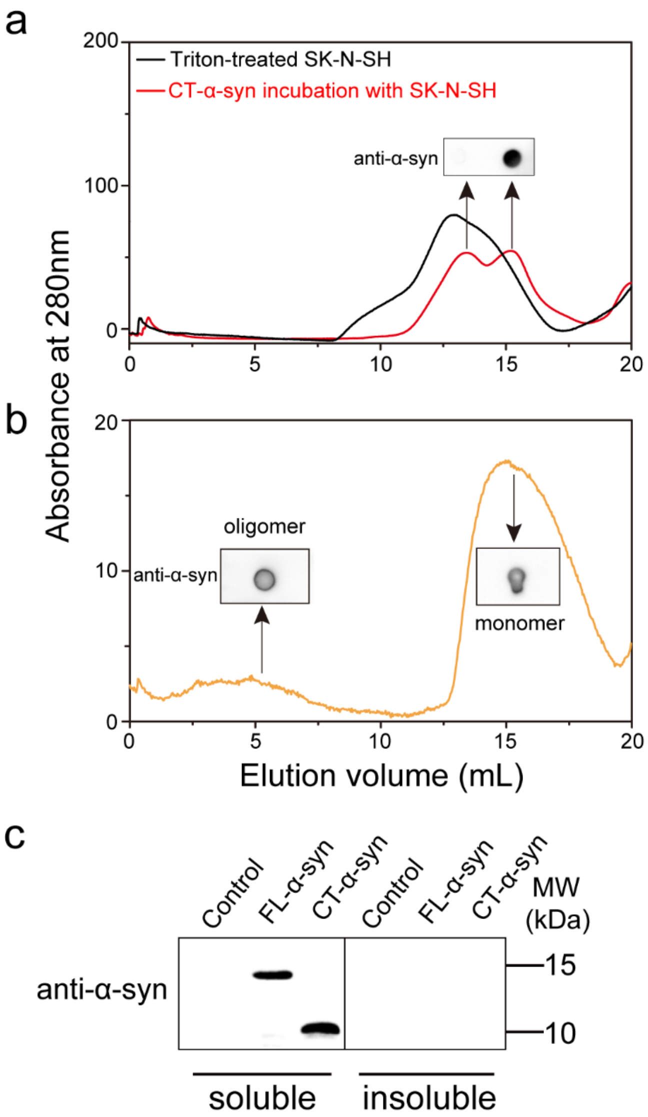

CT-α-syn exists as monomer during 5 h incubation with SK-N-SH-cells. (a) Size-exclusion chromatography of Triton-treated SK-N-SH cells (black line) and CT-α-syn incubated with SK-N-SH cells (red line) for 5 h. (b) Size-exclusion chromatography and dot blot analysis of CT-α-syn oligomers and monomers. CT-α-syn oligomers were generated according to previous studies(1, 2). Briefly, lyophilized CT-α-syn was dissolved in PBS buffer (pH7.4) to a final concentration of ~800 μM and incubated in 37 °C for 22 h without agitation. Fibrils formed during that time were removed by centrifugation at 15,000 *g* for 15 min. CT-α-syn monomers (10 kDa) and small oligomers were removed by filtration through 30 kDa cutoff membrane. The primary antibody recognizes the 1-100 N-terminal residues of α-syn (Abcam, Cat.ab51252). (c) Immunoblots of detergent-soluble and insoluble α-syn after 5 h incubation with cells. Samples prepared as described previously(3, 4). After 5 h incubation, Triton X-100 is added to FL- or CT-α-syn to a final concentration of 1% and incubated on ice for 30 min followed by centrifugation at 15,000 *g* for 15 min at 4 °C. The supernatant is referred to as the soluble fraction. The pellet is then resuspended in loading buffer (2% SDS), washed with PBS, and boiled for 15 min (insoluble fraction).

Supplementary Figure 3

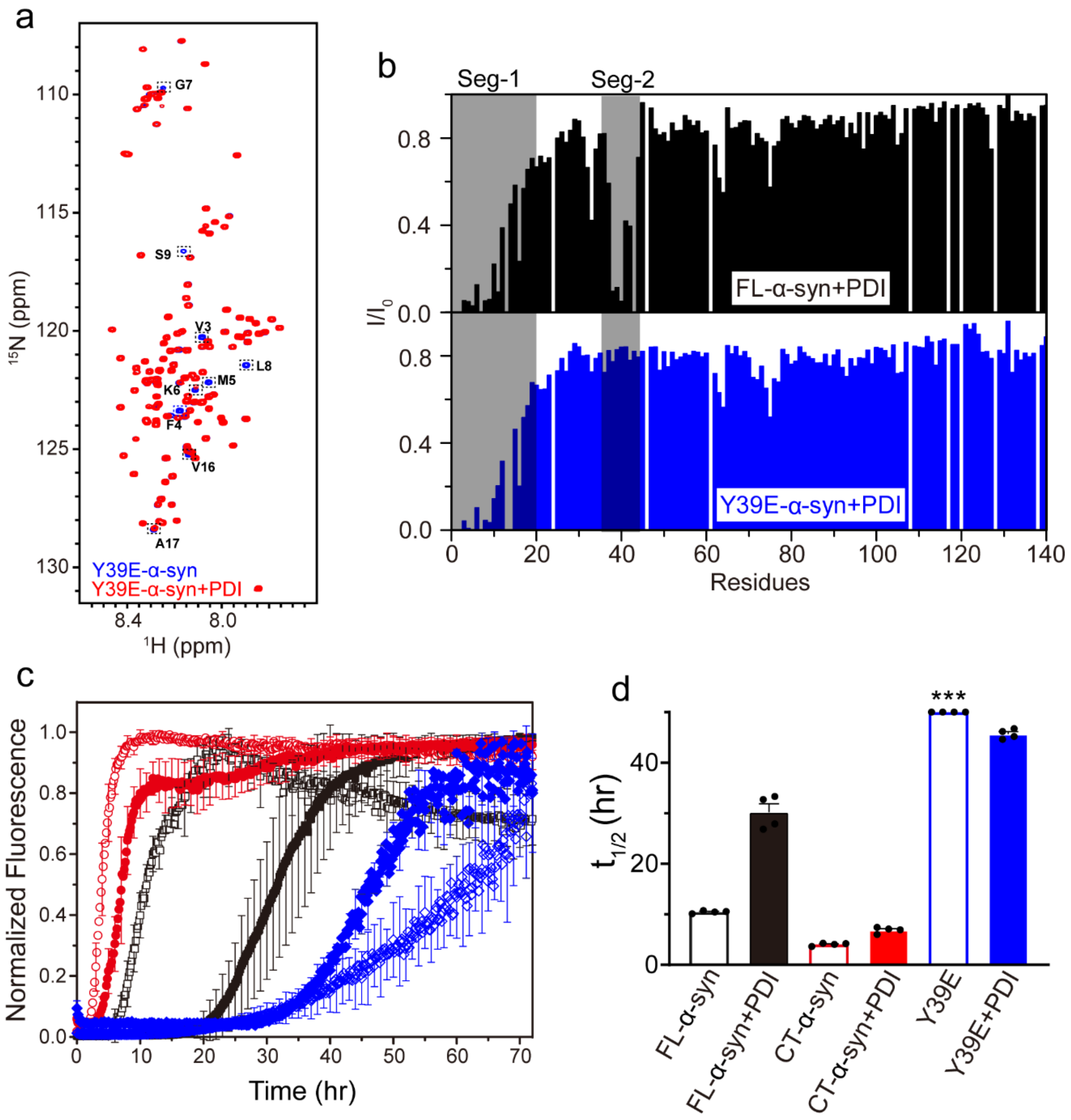

Interaction of Y39E-α-syn with PDI. (a) Overlaid ^{1}H-^{15}N HSQC spectra of 0.3 mM ^{15}N-enriched Y39E-α-syn in the absence (blue) and presence (red) of 0.15 mM PDI. Perturbed residues are surrounded by a dotted box. (b) Residue-resolved NMR signal attenuation (I/I_0) of 0.3 mM FL-α-syn (black bars) and Y39E-α-syn (blue bars) upon adding 0.3 mM PDI. Values<1.0 indicate interaction. Binding regions are colored gray. (c) Thioflavin T fluorescence-monitored aggregation kinetics of Y39E, FL- and CT-α-syn in the absence and presence of equimolar PDI and (d) the half-times of aggregation ($t_{1/2}$). Error bars represent the standard deviation of the mean from four independent experiments [*** indicates slow aggregation (i.e., no plateau)].

Supplementary Figure 4

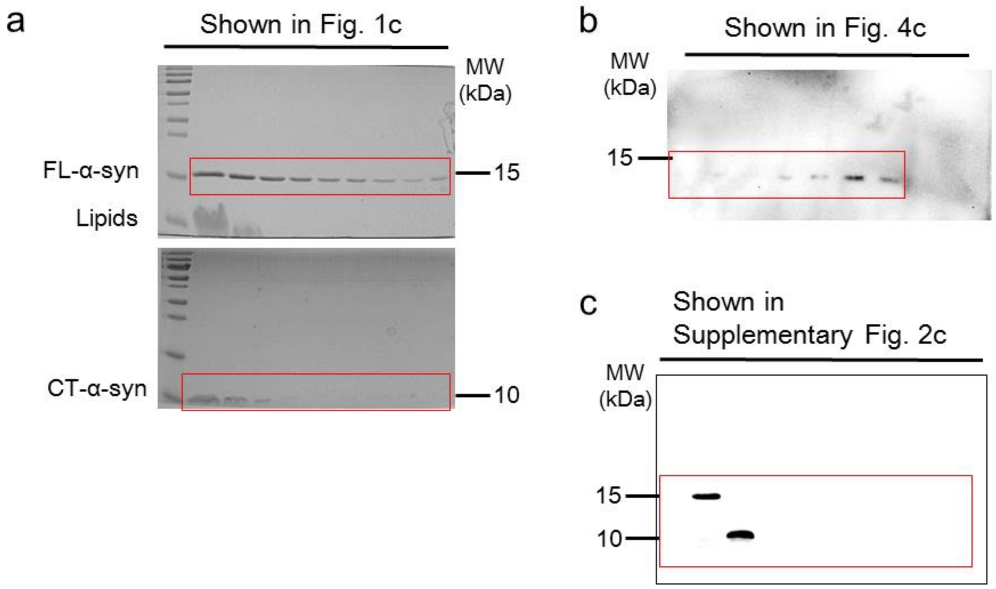

Full gel/blot images. (a) Full gel images correspond to Fig. 1c. (b) Full blot image corresponds to Fig. 4c. (c) Full blot image corresponds to Supplementary Fig. 2c.

Supplementary References

1. Chen SW*, et al.* Structural characterization of toxic oligomers that are kinetically trapped during α-synuclein fibril formation. *Proc. Natl. Acad. Sci. U. S. A.* 112, E1994-2003 (2015).
2. Fusco G*, et al.* Structural basis of membrane disruption and cellular toxicity by α-synuclein oligomers. *Science* 358, 1440-1443 (2017).
3. Dutta D*, et al.* Selective targeting of the TLR2/MyD88/NF-κB pathway reduces α-synuclein spreading in vitro and in vivo. *Nat. Commun.* 12, 5382 (2021).
4. Klucken J, Shin Y, Masliah E, Hyman BT, & McLean PJ. Hsp70 reduces alpha-Synuclein aggregation and toxicity. *J. Biol. Chem.* 279, 25497-25502 (2004).
