# Supplementary material for: C-terminal truncation modulates α-Synuclein’s cytotoxicity and aggregation by promoting the interactions with membrane and chaperone
Source: Commun Biol. 2022 Aug 9;5:798. doi: 10.1038/s42003-022-03768-0 (PMC9363494; doi:10.1038/s42003-022-03768-0)
Supplement: Supplementary file 3 — Description of Additional Supplementary Data [file 42003_2022_3768_MOESM3_ESM.pdf]

Description of Additional Supplementary Files

File name: Supplementary Data 1
Description: Source data Fig. 1

File name: Supplementary Data 2
Description: Source data Fig. 2

File name: Supplementary Data 3
Description: Source data Fig. 4
