# Supplementary material for: C-terminal truncation modulates α-Synuclein’s cytotoxicity and aggregation by promoting the interactions with membrane and chaperone
Source: Commun Biol. 2022 Aug 9;5:798. doi: 10.1038/s42003-022-03768-0 (PMC9363494; doi:10.1038/s42003-022-03768-0)
Supplement: Supplementary file 7 — Reporting Summary [file 42003_2022_3768_MOESM7_ESM.pdf]

nature portfolio

Corresponding author(s): Conggang Li, Zeting Zhang

Last updated by author(s): Jul 1, 2022

Reporting Summary

Nature Portfolio wishes to improve the reproducibility of the work that we publish. This form provides structure for consistency and transparency in reporting. For further information on Nature Portfolio policies, see our Editorial Policies and the Editorial Policy Checklist.

Statistics

For all statistical analyses, confirm that the following items are present in the figure legend, table legend, main text, or Methods section.

n/a	Confirmed	
☐	☒	The exact sample size (*n*) for each experimental group/condition, given as a discrete number and unit of measurement
☐	☒	A statement on whether measurements were taken from distinct samples or whether the same sample was measured repeatedly
☐	☒	The statistical test(s) used AND whether they are one- or two-sided<br>*Only common tests should be described solely by name; describe more complex techniques in the Methods section.*
☒	☐	A description of all covariates tested
☐	☒	A description of any assumptions or corrections, such as tests of normality and adjustment for multiple comparisons
☐	☒	A full description of the statistical parameters including central tendency (e.g. means) or other basic estimates (e.g. regression coefficient) AND variation (e.g. standard deviation) or associated estimates of uncertainty (e.g. confidence intervals)
☐	☒	For null hypothesis testing, the test statistic (e.g. *F*, *t*, *r*) with confidence intervals, effect sizes, degrees of freedom and *P* value noted<br>*Give P values as exact values whenever suitable.*
☒	☐	For Bayesian analysis, information on the choice of priors and Markov chain Monte Carlo settings
☒	☐	For hierarchical and complex designs, identification of the appropriate level for tests and full reporting of outcomes
☒	☐	Estimates of effect sizes (e.g. Cohen's *d*, Pearson's *r*), indicating how they were calculated

Our web collection on statistics for biologists contains articles on many of the points above.

Software and code

Policy information about availability of computer code

Data collection	NMR data were collected on Bruker spectrometers operated with TOPSPIN 3.5.
Data analysis	NMR data were processed with NMRpipe and analyzed with Sparky.

For manuscripts utilizing custom algorithms or software that are central to the research but not yet described in published literature, software must be made available to editors and reviewers. We strongly encourage code deposition in a community repository (e.g. GitHub). See the Nature Portfolio guidelines for submitting code & software for further information.

Data

Policy information about availability of data

All manuscripts must include a data availability statement. This statement should provide the following information, where applicable:

- Accession codes, unique identifiers, or web links for publicly available datasets
- A description of any restrictions on data availability
- For clinical datasets or third party data, please ensure that the statement adheres to our policy

The data that support the findings of this study are available from the corresponding authors upon request.

Field-specific reporting

Please select the one below that is the best fit for your research. If you are not sure, read the appropriate sections before making your selection.

☒ Life sciences ☐ Behavioural & social sciences ☐ Ecological, evolutionary & environmental sciences

For a reference copy of the document with all sections, see nature.com/documents/nr-reporting-summary-flat.pdf

Life sciences study design

All studies must disclose on these points even when the disclosure is negative.

Sample size	Dynamic light scattering was used to determine the particle size of liposomes.
Data exclusions	No data was excluded from the analysis.
Replication	The experiments were replicated to assure reproducibility of the results. The number of independent replicates for each experiment are indicated in the corresponding figure captions. All attempts at replication were successful.
Randomization	The experiments were not randomized, conformed to the established procedures in the field.
Blinding	It was not blind to appoint the investigators during experiments and outcome evaluation, conformed to the established procedures in the field.

Reporting for specific materials, systems and methods

We require information from authors about some types of materials, experimental systems and methods used in many studies. Here, indicate whether each material, system or method listed is relevant to your study. If you are not sure if a list item applies to your research, read the appropriate section before selecting a response.

Materials & experimental systems

n/a	Involved in the study	
☐	☒	Antibodies
☐	☒	Eukaryotic cell lines
☒	☐	Palaeontology and archaeology
☒	☐	Animals and other organisms
☒	☐	Human research participants
☒	☐	Clinical data
☒	☐	Dual use research of concern

Methods

n/a	Involved in the study	
☒	☐	ChIP-seq
☒	☐	Flow cytometry
☒	☐	MRI-based neuroimaging

Antibodies

Antibodies used	Rabbit anti-Cytochrome c Abcam Cat#ab133504/Rabbit anti-Alpha-synuclein Abcam Cat#ab51252, 1:1000 for Western blot
Validation	Rabbit anti-Cytochrome c Abcam Cat#ab133504/Rabbit anti-Alpha-synuclein Abcam Cat#ab51252, validated by Abcam

Eukaryotic cell lines

Policy information about cell lines

Cell line source(s)	SK-N-SH cells were purchased from Procell (CL-0214)
Authentication	The authenticity of the cells was provided by Procell and the American Type Culture Collection upon purchase.
Mycoplasma contamination	Mycoplasma-free cultures were used for the experiments.
Commonly misidentified lines (See ICLAC register)	No commonly misidentified cells were used.
